# Digital Health Literacy in Patients With Common Chronic Diseases: Systematic Review and Meta-Analysis

**DOI:** 10.2196/56231

**Published:** 2025-08-25

**Authors:** Hadeel Zaghloul, Kareem Fanous, Lina Ahmed, Maryam Arabi, Sanish Varghese, Sara Omar, Yousef Al-Najjar, Rayane El-Khoury, Jamie Gray, Amine Rakab, Thurayya Arayssi

**Affiliations:** 1 Department of Internal Medicine, Weill Cornell Medicine in Qatar, Al Luqta Street, PO Box 24144, Doha, Qatar, 974 66107560

**Keywords:** chronic disease management, eHealth literacy scale, diabetes mellitus, hypertension, rheumatoid arthritis, digital health literacy, chronic disease, eHealth literacy, systematic review, meta-analysis, literature review, diabetes, eHEALS, demographic, socioeconomic, database, self-management, self-care, meta-regression analysis

## Abstract

**Background:**

Digital health technology (DHT) plays an increasingly vital role in managing chronic diseases by enabling patients to actively manage their health. These tools have been shown to improve self-management and adherence to medical advice. However, for DHT to be fully effective, patients with chronic conditions must be digitally literate. The eHealth Literacy Scale (eHEALS), an 8‑item tool with scores ranging from 8 to 40, was developed to assess individuals’ perceived ability to find, evaluate, and apply digital health information. Assessing patients’ digital health literacy (DHL) and understanding the factors influencing it are essential for improving the accessibility and usability of health resources.

**Objective:**

This study aimed to assess DHL in patients with diabetes mellitus (DM), hypertension, and rheumatoid arthritis (RA) through a systematic review and meta‑analysis using eHEALS. We sought to determine average DHL scores, examine demographic and socioeconomic factors influencing DHL, and explore its impact on disease management to inform future strategies for enhancing DHL and improving chronic disease outcomes.

**Methods:**

Following PRISMA (Preferred Reporting Items for Systematic Reviews and Meta-Analyses) guidelines, we conducted a systematic review across 7 databases (PubMed, SCOPUS, Embase, ERIC, CINAHL, Library Literature and Information Science Index, and Google Scholar) from inception to August 14, 2022, with an updated search in October 2024. Eligible studies included adults (≥18 years) with DM, hypertension, or RA who reported DHL data using eHEALS (8-40) and were original research published in English. Exclusion criteria included studies involving participants younger than 18 years, reviews, meta‑analyses, studies not addressing the target diseases, or non‑English publications. Study quality was evaluated using the Newcastle‑Ottawa Scale (NOS).

**Results:**

Eight studies involving 2527 participants were included. The pooled mean eHEALS score was 27.03 (95% CI 25.08-28.98), indicating high overall DHL. Stratified by disease, scores were higher for DM (27.79) and hypertension (28.48) but lower for RA (24.74). Quality assessment indicated a high standard of included studies. Factors influencing DHL included age, education, employment, and perception of the internet as a health resource. Due to the limited number of studies, meta‑regression analysis could not be performed.

**Conclusions:**

DHL is critical for individuals with chronic conditions, empowering them to make informed decisions and manage their health effectively. However, the scarcity of studies limits comprehensive analysis of DHL determinants. While the internet offers abundant health information, unequal DHL and health skills remain barriers. More inclusive research is needed to fully understand DHL’s impact on health outcomes and mitigate disparities, ensuring equitable access to digital health resources and improving disease management.

## Introduction

Digital health technology (DHT; such as mobile health devices, wearables and sensors, and remote monitoring) has the potential to revolutionize the management of chronic diseases by empowering patients to take a more active role in their own care, enhancing disease monitoring, optimizing treatment and adherence, and reducing health care costs. Individuals are increasingly using electronic tools to take charge of their health management [1,2]. In addition, there is evidence that programs delivered through DHT can lead to better self-management behaviors and adherence to medications and lifestyle recommendations [3]. Wearable devices, such as smartwatches and activity trackers, can help patients monitor their physical activity, sleep patterns, and vital signs [4], while mobile apps [5] and internet-based platforms [6] can provide access to educational resources, support groups, and remote consultations with health care professionals. However, to fully realize the benefits of DHT, people with chronic diseases need to be digitally literate and confident in using these tools. This requires not only access to DHT, but also education and support to help patients navigate and use these tools effectively. Digital health literacy (DHL) has been described as “the capacity to actively search for, locate, comprehend, and evaluate health information obtained from electronic sources, and subsequently apply the acquired knowledge to manage or resolve a health issue” [7]. Consequently, to enhance accessibility and usability of the available resources, it becomes imperative to evaluate a patient’s DHL and ascertain the factors that influence it.

Norman and Skinner [7] have developed a valid and reliable tool to assess DHL known as the eHealth Literacy Scale (eHEALS). The eHEALS is an 8-item scale with a cumulative score ranging from 8 to 40. The sum of individual item scores reflects a person’s level of self-reported eHealth literacy. Higher scores on this scale indicate a more profound capability in navigating digital health resources. The eHEALS has been validated in many languages [8-12] and for use in older adults [13,14], demonstrating its applicability across diverse demographic segments. eHEALS offers self-reported insights into an individual’s familiarity, comfort level, and perceived proficiency in using the internet for accessing health-related information [15]. A score of ≥26 is accepted as the cutoff for high DHL using the eHEALS [16].

As the burden of chronic diseases continues to rise, especially in low- and middle-income countries, the cost of their management continues to grow [17]. In today’s era of rapidly advancing technology and widespread digital connectivity, the intersection of health care and digital literacy has become crucial [18]. DHL plays a pivotal role in empowering people with chronic diseases to make informed decisions about their health and well-being. As these individuals navigate a digital landscape filled with health apps, internet-based resources, telemedicine platforms, and eHealth records, possessing adequate DHL is fundamental for optimizing their ability to manage their conditions, communicate with health care providers, and ultimately enhance their overall quality of life. Among individuals with chronic illnesses, adopting health-enhancing behaviors can contribute to diminishing prevalence rates and reducing expenses related to health management [19]. Studies have consistently demonstrated that higher health literacy results in more health-enhancing behaviors and therefore reduction in health-related costs [20]. Currently, digital resources are one of the most important sources for health-related data. In fact, before consulting a physician, internet users seek information about their health status and subsequently revisit the internet to seek expert advice or recommendations following the consultation [21]. Thus, improving DHL is not about improving individual health outcomes, but also about reducing the broader economic burden of chronic diseases.

Researchers have made efforts to solidify the foundation of evidence supporting the design and advancement of DHTs [22]. Regrettably, these valuable reservoirs can only yield benefits if patients possess sufficient literacy to use them. Understanding DHL levels in people with chronic diseases is thus crucial to inform clinical decision-making, personalized treatment strategies, and the development of effective digital health interventions. Importantly, DHL has been found to be modifiable, and multiple studies have demonstrated its enhancement among older individuals and those managing chronic health conditions [23].

We therefore sought to conduct a systematic review and meta-analysis of DHL using eHEALS in people with 3 common chronic diseases that require self-management: diabetes mellitus (DM), hypertension, and rheumatoid arthritis (RA). As a primary outcome, we aimed to comprehensively characterize DHL among people with common chronic diseases and to estimate the pooled mean of the eHEALS scores among them. In addition, we aimed to identify demographic and socioeconomic factors that influence DHL levels in this population. Furthermore, the study strives to determine the impact of DHL on patient outcomes. Through this review, we seek to contribute to the body of knowledge that informs future interventions to improve DHL and thereby health outcomes in chronic disease management.

## Methods

### Data Sources and Search Strategy

This systematic review was conducted in accordance with PRISMA (Preferred Reporting Items for Systematic Reviews and Meta-Analyses) guidelines [24] (Checklist 1) and used a predefined protocol registered with PROSPERO (International Prospective Register of Systematic Reviews; CRD42023467462). Our search strategy was developed with the assistance of a medical librarian (JG). We conducted a systematic literature review of 7 databases (PubMed, SCOPUS, Embase, ERIC, CINAHL, Library Literature and Information Science Index, and Google Scholar) from their inception till the date the search was executed on August 14, 2022. An updated search was performed in October 2024 to ensure no recent studies had been published since the initial search. The study aimed to identify studies examining DHL using the eHEALS tool [7] in adult people with 3 common chronic diseases that require self-management: DM, hypertension, and RA. Inclusion criteria were (1) studies conducted in adult patients (≥18 years) with DM, hypertension, or RA; (2) studies reporting on DHL; (3) studies using eHEALS to measure DHL on a scale from 8 to 40; (4) studies in English language; and (5) all original research studies. Exclusion criteria were (1) studies conducted in people <18 years; (2) literature reviews or systematic reviews; (3) studies not reporting specifically on DM, hypertension, or RA; and (4) studies in languages other than English. Details on the search strategy are available in Multimedia Appendix 1. Briefly, our search strategy combined synonyms for DHL with synonyms for the 3 disease conditions. The search was limited to English articles only as translation was not possible. In addition, we manually reviewed reference lists of all relevant publications to ensure inclusion of all relevant articles.

### Study Selection

A total of 2 out of 6 reviewers (KF, LA, MA, SV, SO, and YA) independently assessed the titles and abstracts of each of the identified references (N=386) to determine initial eligibility. Studies were determined to be eligible, noneligible, or uncertain. Full texts were retrieved for 92 references and were independently examined by 2 reviewers to determine eligibility. In case of uncertainty or disagreement at each stage, consensus was reached by consulting a third party (HZ and AR).

### Data Extraction and Quality Assessment

A data extraction form was developed in Covidence for the extraction of data from eligible research papers and included general study characteristics, population included, methods, and outcome measures. The data extraction form was piloted prior to full data extraction using 3 studies selected for initial testing to ensure that the extraction template was clear, comprehensive, and consistently applied across reviewers. Two reviewers independently extracted data from each study, and disagreements were reconciled by consensus with a third party (HZ).

The Newcastle-Ottawa Scale (NOS) was used to evaluate the quality of included studies. The NOS uses 8 assessment items for quality appraisal that include “selection” (assesses whether study participants are selected in an unbiased manner), “comparability” (examines whether studies control for confounding factors), and “outcome” (evaluates the measurement of outcomes and the adequacy of follow-up) [25]. According to the NOS score standard, cross-sectional studies could be classified as low-quality (scores of 0‐4), moderate-quality (scores of 5‐6), and high-quality (scores ≥7). Two investigators (HZ and AR) conducted the quality assessment independently. In case of differing opinions, a consensus was reached by discussion between investigators.

### Data Synthesis and Statistical Analysis

We conducted a meta-analysis on the reported mean eHEALS score. Every score was taken as a point of analysis. Studies reporting more than one score for different chronic diseases [23] were taken as a separate outcome. Studies with no reported SD for the mean eHEALS score were imputed using the median value of the available SDs. Pooled mean estimates for eHEALS scoring stratified by disease type were calculated. Cochran Q statistic was used to assess the presence of heterogeneity in effect size. *I*^2^ was used to measure the magnitude of between-study heterogeneity due to true differences in effect size rather than chance. The 95% CI of the distribution of true eHEALS score was used to describe the distribution of true effect sizes. Meta-analyses were conducted in Stata Statistical Software: Release 18 (StataCorp LLC) [26], using the random-effects (REML [Restricted Maximum Likelihood) model. A meta-regression was not possible due to the small number of included studies.

## Results

### Selection and Inclusion of Studies

**Figure 1.**
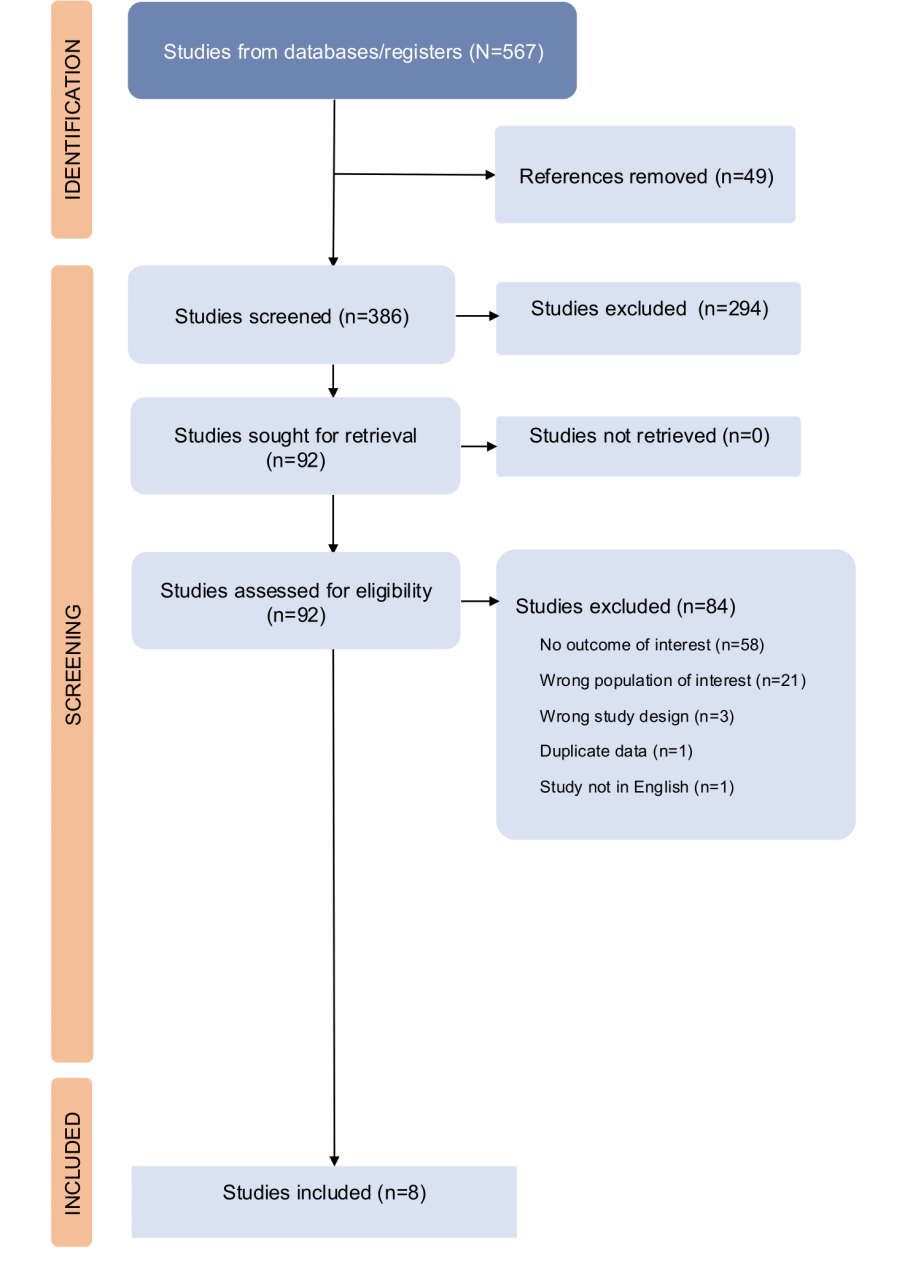
Flowchart of article selection for the systematic review of DHL in patients with diabetes mellitus, hypertension, and rheumatoid arthritis, adapted from the PRISMA (Preferred Reporting Items for Systematic reviews and Meta-Analyses) 2009 guidelines.

In our study, we first meticulously evaluated a range of studies to assess their relevance and quality, following a rigorous selection process to identify the most pertinent research for our analysis. We screened a total of 386 titles and abstracts for inclusion in the study after removing duplicates. We excluded most studies (n=294) at this stage, leaving 92 studies for full-text examination. During the full-text screening stage, 84 studies were excluded. Figure 1 details reasons for full-text exclusion. Notably, although using eHEALS, 2 studies diverged from the standard scoring methods (results ranging from 8 to 40) [27,28]. Despite efforts to clarify these deviations through email communication with the authors, the lack of response necessitated their exclusion from our data synthesis. A total of 8 studies were included in the systematic review and meta-analysis.

### Study Characteristics

A total of 567 studies were retrieved, which were deduplicated using EndNote (Clarivate) and Covidence. A total of 163 references were identified as duplicates and removed (132 in EndNote and 31 in Covidence). Remaining references (n=404) were imported into Covidence for screening, of which another 18 were manually identified as duplicates, leaving a total of 386 articles for screening. Figure 1 shows the PRISMA schematic for study selection.

This review included 8 studies involving 2527 individuals [10,11,23,29-33]. Table 1 details the main characteristics of these studies. All the studies, conducted between 2011 and 2022, were cross-sectional. In addition, 2 of these studies used mixed methods [11,32], combining qualitative and quantitative approaches. A total of 4 out of 8 studies were in people with DM [11,29,30,32], 2 in people with RA [10,33], 1 in people with hypertension [31], and 1 in people with both DM and hypertension [23]. Half of the studies were conducted on participants of Asian ethnicity [23,29-31], 2 in Caucasians [10,32], 1 study in Hispanics [11], and 1 included participants from 18 countries across Europe, Asia, and South America [33]. The study sample size ranged from 20 to 1609. In total, 3 studies had less than 100 participants [11,23,32]. The majority of studies had between 100 and 250 participants [10,29-31], and only 1 study [33] had more than 250 participants.

**Table 1.** Characteristics of included studies.

Sources	Country	Study objectives	Study design	Percentage of females	Age of participants	Disease	Sample size	eHEALS^a^ score
Aponte et al, 2017 [11]	United States	To explore the experiences of older Hispanics with T2DM^b^ in using the internet for DM^c^ management.	Convergent mixed methods design: quantitative and qualitative	50%	74, range 68‐86 (5.59)	DM	20	Mean ( SD ): 22.35 (12.9)
Guo et al, 2021 [29]	Taiwan	Examine online information-seeking behavior and mobile health app usage, investigate the factors related to mobile DHL^d^ in Taiwanese patients with T2DM, and relationship between DHL, mobile health literacy, and health outcomes.	Cross-sectional study	34.1%	Mean (SD): 44.58+−11.02 Median (IQR): Not reported	DM	249	Mean (SD): 30.16 (5.41), Range: 8‐40
Kim et al, 2018 [30]	South Korea	Examine the association among DHL, perceived benefits, self-efficacy, and health-promoting behaviors in patients with T2DM, and identify factors that affect health-promoting behaviors.	Cross-sectional study	46.3%	Mean (SD): 61.29 (10.99)	DM	203	Mean (SD): 27.15 (5.43), Range: 8‐40
Makowsky et al, 2022 [23]	Canada	Describe perceived DHL and explore the extent to which it is associated with sociodemographic, health status, and technology use variables in a subset of South Asian Canadians.	Cross-sectional study	55.1%	Mean (SD): 39.9 (14.8)	DM and HTN^e^	Diabetes: 42 HTN: 55;	Diabetes Mean (SD): 28.76 (6.25) HTN: 26.95 (8.35)
Rojanasumapong et al, 2021 [31]	Thailand	To explore the internet usage and DHL among adults aged 60 and older with HTN and associations between DHL and blood pressure control	Cross-sectional study	57.3%	Mean (SD): 67 (5.23)	HTN	110	Mean ( SD ): 29.6 (4.15)
Sjöström et al, 2021 [32]	Sweden	Explore online COVID-19 information acquisition experiences among persons with T2DM and varying DHL.	Qualitative data from in-depth interviews and free-text answers from questions added to the eHEALS	48.3%	Median: 73 Range: 41‐82	DM	58	Mean: 26 .67
Taylor et al, 2021 [33]	18 countries across Europe, Asia, and South America	Assessing the impact of inadequate response to DMARDs^f^ on treatment satisfaction, disease outcomes, and patient perspectives related to RA^g^ disease management.	Cross-sectional study	84.2%	Mean (SD): 58.4 (13.1)	RA	1601	Mean ( SD ): 21.3 (8.4)
Rosalie van der Vaart, 2011 [10]	Netherlands	To assess the internal consistency, construct validity, and predictive validity of a Dutch translation of the eHEALS in 2 populations.	Cross- sectional study	37%	Mean (SD): 52 (11)	RA	189	Mean ( SD ): 28.2 (5.9)

a eHEALS: eHealth Literacy Scale.

b T2DM: type 2 diabetes mellitus.

c DM: diabetes mellitus.

d DHL: digital health literacy.

e HTN: hypertension.

f DMARDs: disease-modifying antirheumatic drugs.

g RA: rheumatoid arthritis.

### Meta-Analysis Findings

**Figure 2.**
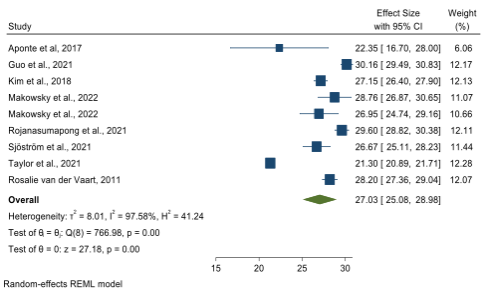
Pooled mean estimates for eHealth Literacy Scale scoring [10,11,23,29-33]. REML: Restricted Maximum Likelihood.

**Figure 3.**
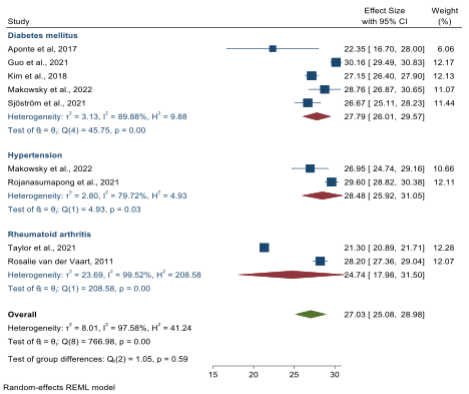
Pooled mean estimates for eHealth Literacy Scale scoring by disease type [10,11,23,29-33]. REML: Restricted Maximum Likelihood.

In our meta-analysis, we synthesized data from 8 studies [10,11,23,29-33] to estimate the level of DHL across various patient groups, taking into account different disease conditions. Our synthesis yielded a total of 9 measures for analysis, since 1 study included 2 distinct populations [23]. We calculated an overall pooled mean eHEALS score of 27.03 (95% CI 25.08‐28.98), indicating a high level of DHL (Figure 2). Stratifying the analysis by disease type (Figure 3), we found that in the DM group, the pooled mean eHEALS score was 27.79 (95% CI 26.01‐29.57). In the hypertension group, this score was 28.48 (95% CI 25.92‐31.05). For the RA group, the score was 24.74 (95% CI 17.98‐31.50). In summary, our meta-analysis reveals a generally high level of DHL across different patient groups, with some variation observed based on the type of disease.

### Quality Assessment of Studies

For the quality assessment, the quality score of the included studies, assessed by the NOS, ranged from 7 to 9 (mean 8, SD 0.93 score; Table 2).

**Table 2.** Quality assessment of included studies using the Newcastle-Ottawa Scale.

Study	Selection	Comparability	Outcome	Total
Aponte et al [11]	3/5	2/2	2/3	7/10
Guo et al [29]	5/5	2/2	2/3	9/10
Kim et al [30]	5/5	2/2	2/3	9/10
Makowsky et al [23]	4/5	2/2	2/3	8/10
Rojanasumapong et al [31]	4/5	2/2	2/3	8/10
Sjöström et al [32]	4/5	2/2	1/3	7/10
Taylor et al [33]	5/5	0/2	2/3	7/10
Rosalie van der Vaart [10]	5/5	2/2	2/3	9/10

## Discussion

### Principal Findings

The findings from our study indicate that in people with 3 common chronic diseases that require self-management, overall DHL was found to be high. Upon stratification of the results by disease type, it becomes evident that the mean DHL scores for people with DM and hypertension remained notably elevated, while a significant departure is observed in the case of people with RA, indicating that patients with RA have a lower DHL score. Due to the small number of eligible studies, a meta-regression was not possible, and therefore, we could not draw any conclusions regarding the factors that affected DHL in this population. It is also important to highlight that only 2 of the eligible studies [29,30] aimed to investigate DHL or factors associated with it, thus making it difficult to draw conclusions regarding factors associated with DHL.

Our analysis revealed notable differences in DHL among patients with RA compared to those with DM and hypertension. These variations may stem from the unique disability profile associated with RA, including its profound impact on quality of life, work-related factors, and the educational levels prevalent within this patient group. It is plausible that the unique disability profile associated with RA may underpin these differences in DHL between the study populations. This finding is in line with previous reports of a digital divide between people with disabilities and the rest of the population, where there is a lower level of internet use in people with disabilities [34]. The lower use of the internet in persons with disabilities is largely explained by income level, where those with disabilities tend to be generally poorer than those without. Individuals with disabilities continue to use the internet less frequently, even after accounting for socioeconomic variables such as education, income, and employment [34]. Taylor et al [33] reported that there was a significant impairment in the quality of life of their participants (people with RA). Moreover, RA significantly influenced the work lives of patients, with 11.9% opting for early retirement because of RA, and 6% facing unemployment as a result of the condition. Interestingly, retirement has been shown to reduce the computer literacy and the frequency of internet use [35]. Although not conclusive, this could indicate that the daily functioning of the participants, including their internet usage, may have been significantly impaired and hence affecting DHL. Education level is another factor associated with DHL [36]. In our review, the studies focusing on patients with RA [10,33] revealed that approximately one-third had attained education beyond secondary school, compared to more than half as reported in studies of other patient groups. This could also explain the difference in DHL levels observed when the studies are stratified by disease type. However, given the limited number of studies, it is essential to note that these are observed patterns requiring further formal validation. If confirmed, the implications for a lower DHL score among patients with RA could be significant, as it may hinder their ability to effectively engage with digital health resources and access reliable health information. This could contribute to poorer disease management, reduced adherence to treatment plans, and ultimately worse health outcomes. In addition, given the increasing reliance on digital platforms for health care delivery, lower DHL may exacerbate existing health disparities, particularly for individuals with lower socioeconomic status or educational attainment. Therefore, recommendations could include tailored digital health education programs, targeted socioeconomic support, workplace and retirement considerations, health care provider involvement, and further research and policy development. These interventions could help bridge the digital divide for patients with RA, enhancing their ability to manage their condition effectively and improving overall health outcomes. Our findings emphasize the need for further research to unravel the nuances of DHL, with the primary aim of investigating DHL and its associated factors within diverse chronic disease populations.

In a broader context, the findings of individual studies in our review contribute to the ongoing debate about demographic and socioeconomic factors influencing DHL [37]. Whereas some studies found no difference in DHL between younger and older individuals with chronic diseases [11], others found DHL to be higher in younger age [23,30]. Interestingly, Aponte et al [11] found that DHL was much lower in men compared to women (average DHL in men=13.85), not in line with previous findings that there are no gender differences for DHL [36]. However, this result could be attributed to the small sample size used in their study, potentially explaining the deviation from earlier findings. Other factors that were found to be associated with higher DHL were higher education, being used, cohabitation, positive perception of the internet as a source of medical information, and longer time spent on the internet [23,30]. In addition, our review revealed that one study [29] reported a negative correlation between DHL and the duration of diabetes among patients with DM. Compared to our study findings, a recent systematic review examining the sociodemographic determinants of DHL [36] identified a negative impact of age on DHL, particularly among older adults, while gender showed no significant influence. In addition, they indicated that higher educational levels, increased income, and more significant social support were associated with higher DHL levels. However, it is important to note that this systematic review included studies involving both individuals with and without chronic conditions, where factors such as disease burden, health care needs, and digital engagement may differ significantly and thus affect the generalizability of these findings.

Noticeable disparities persist among individuals in terms of their levels of DHL, as well as their proficiency in electronic skills and familiarity with internet usage. These discrepancies have been found to be closely tied to socioeconomic status and the level of independence in using digital tools [36,38]. Consequently, these factors contribute to the existence of social health disparities and suboptimal health results [36,39]. Despite its growing significance, there has been a lack of comprehensive exploration into the demographic, socioeconomic, and technological factors that influence eHealth literacy up until now. As the DHT field continues to expand and explore new avenues for customized interventions [40], ensuring technological advancements enhance rather than exacerbate existing health disparities is critical. Like any tool, the effectiveness of digital health solutions depends on appropriate usage, accessibility, and the culturally sensitive and skill-appropriate resolution of technology-related obstacles.

Middle-aged and elderly individuals, particularly in developing countries, may lack the capacity and habit to independently and persistently use digital chronic disease management systems, leading to limitations in data availability. This challenge is closely tied to DHL, which plays a crucial role in the effective adoption and use of digital health technologies. A recent systematic review assessing DHL found that the average eHealth literacy score was significantly lower than the passing level [41]. Notably, older adults in developing countries exhibited even lower eHealth literacy scores, further reinforcing the digital divide. Key demographic factors such as gender, age (80+years), living alone, and lack of a spouse were also associated with lower eHealth literacy, highlighting the vulnerability of certain subgroups. Given this, we recognize that lower DHL contributes to the underuse of digital chronic disease management tools, impacting the completeness of relevant data in our study. This finding underscores the urgent need for interventions that enhance digital literacy among older populations, such as targeted education programs, simplified user interfaces, and support systems tailored to their needs.

In the broader exploration of DHL among individuals managing chronic diseases, an essential facet to investigate is whether DHL exerts an influence on health outcomes. Regrettably, most studies within our review did not delve into the potential association between DHL and disease outcomes. Rojanasumapong et al [31] was the only study included in our review to explore the effect of DHL on health outcomes and could not establish a statistically significant correlation between blood pressure control and DHL in individuals with hypertension. These findings emphasize a critical gap in the current body of research and underscore the imperative need for future studies to elucidate whether improved DHL indeed translates into enhanced health outcomes for the patient. This represents a pivotal avenue for further exploration within the field of DHL in the context of chronic disease management.

### Limitations

While our findings contribute meaningful insights into the interplay between DHL and chronic disease management, the scope of our analysis was bound by specific parameters that future studies may wish to expand. First, we focused on only 3 chronic diseases in order to define our study population comprehensively. However, this limited scope may not be fully representative of DHL in the context of other chronic conditions that require self-management. In addition, our meta-analysis revealed a small study effect due to the limited number of studies included. The small sample size also precluded the possibility of conducting a meta-regression analysis to gain deeper insights into the various factors influencing DHL.

Second, it is crucial to highlight that eHEALS measures individuals’ confidence and comfort in using the internet for health-related information, rather than their computer skills. Because the data collected rely on individuals’ personal perceptions, there is a potential for them to either underestimate or overestimate their actual internet knowledge and abilities. While eHEALS is reliable, its associations with age, education, and internet usage are weak [10]. Consequently, future studies should include objective measures to assess internet proficiency, as previous research recommended [10]. In this context, we suggest using an improved version of eHEALS that examines a broader range of skills, including computer and internet usage, data retrieval, search strategy development, and information evaluation. Validating these new components, such as internet search competence and direct knowledge, in subsequent studies is necessary for the tool’s fairness and accuracy.

Shifting to the generalizability of the findings, our results underscore the fact that DHL has been insufficiently explored among patients with chronic diseases from more diverse ethnic backgrounds. A majority of the studies we identified focused on Asian populations, with limited representation from other ethnicities. Consequently, there’s a compelling need for additional research on DHL among patients with chronic diseases, especially within ethnically diverse patient groups.

### Conclusions

Our study highlights the crucial role of DHL in empowering individuals to make informed decisions about their health in the digital age. While the internet serves as a valuable resource, it also harbors misinformation, emphasizing the importance of health care providers guiding patients in discerning reliable sources. Disparities in DHL persist, tied to socioeconomic status and digital proficiency, contributing to social health disparities. We have identified various factors influencing DHL, such as age, education, and employment. These findings underscore the need for further exploration, especially in ethnically diverse patient groups, to ensure tailored interventions. Moreover, the impact of DHL on health outcomes remains a crucial avenue for research, as it could significantly affect patient well-being. Recognizing the limitations of the current assessment tools, future studies should adopt a more comprehensive approach to evaluate internet proficiency. As the field of DHT continues to evolve, it becomes imperative to ensure that technology usage addresses existing health disparities and empowers individuals to navigate the digital landscape effectively.

## Supplementary material

10.2196/56231 Multimedia Appendix 1 Search strategy.

10.2196/56231 Checklist 1 PRISMA checklist.

## Abbreviations

DHL: digital health literacy
DHT: digital health technology
DM: diabetes mellitus
eHEALS: eHealth Literacy Scale
NOS: Newcastle-Ottawa Scale
PRISMA: Preferred Reporting Items for Systematic Reviews and Meta-Analyses
PROSPERO: International Prospective Register of Systematic Reviews
RA: rheumatoid arthritis
REML: Restricted Maximum Likelihood
