# Supplementary material for: Digital Health Literacy in Patients With Common Chronic Diseases: Systematic Review and Meta-Analysis
Source: J Med Internet Res. 2025 Aug 25;27:e56231. doi: 10.2196/56231 (PMC12377790; doi:10.2196/56231)
Supplement: Multimedia Appendix 1 [file jmir-v27-e56231-s001.pdf]

DHL searches
Date executed – August 14, 2022

Database	Results	Platform
PubMed	70	PubMed
SCOPUS	102	Scopus
Embase	268	Ovid
ERIC	13	Ovid
CINAHL	37	Ebsco
Library Literature and Information Science Index	2	Ebsco
Google Scholar	74	Google

Limiting to English was decided as translation not possible
Deduplication done by using Endnote and Covidence
Total Retrieved - 567
Duplications removed – 132 (Endnote) 31(Covidence)
Final citations for review from Endnote- 435
Final citations for review from Covidence - 404

Final Search Strategies

Database	Strategy
PubMed	"health literacy"[MESH] AND ("digital"[Title/Abstract] OR "electronic"[Title/Abstract] OR "online"[Title/Abstract] OR "eHEALS"[Title/Abstract] OR "ehealth literacy scale"[Title/Abstract]) AND ("Arteriosclerosis"[MeSH Terms] OR "Arteriolosclerosis"[MeSH Terms] OR "Arteriosclerosis"[Title/Abstract] OR "Arteriolosclerosis"[Title/Abstract] OR "arthritis, rheumatoid"[MeSH Terms] OR "rheumatoid arthritis"[Title/Abstract] OR "Diabetes Mellitus"[MeSH Terms] OR "Glucose Intolerance"[MeSH Terms] OR "Diabetes Mellitus"[Title/Abstract] OR "type 1 diabetes"[Title/Abstract] OR "type one diabetes"[Title/Abstract] OR "type 2 diabetes"[Title/Abstract] OR "type two diabetes"[Title/Abstract] OR "Glucose Intolerance"[Title/Abstract] OR "Hypertension"[MeSH Terms] OR "Hypertension"[Title/Abstract] OR "high blood pressure"[Title/Abstract]) AND (english[Filter])
SCOPUS	(TITLE-ABS (arteriosclerosis OR arteriolosclerosis OR "rheumatoid arthritis" OR "Diabetes Mellitus" OR "type 1 diabetes" OR "type one diabetes" OR "type 2 diabetes" OR "type two diabetes" OR hypertension OR "high blood pressure")) AND ((TITLE-ABS (eheals OR "ehealth literacy scale")) OR (TITLE-ABS ("health literacy" AND (online OR digital OR electronic)))) AND (LIMIT-TO (LANGUAGE , "English"))
Embase	Embase <1974 to 2022 Week 32>

	1 "health literacy".ti,ab. 14958<br>2 health literacy/ or ehealth literacy/ 16518<br>3 1 or 2 20392<br>4 online.ti,ab. 247460<br>5 digital.ti,ab. 197811<br>6 electronic.ti,ab. 371270<br>7 4 or 5 or 6 792944<br>8 3 and 72965<br>9 eHEALS.ti,ab. 175<br>10 "ehealth literacy scale".ti,ab. 159<br>11 8 or 9 or 10 3045<br>12 arteriolosclerosis.ti,ab. 768<br>13 exp arteriolosclerosis/943<br>14 exp arteriosclerosis/ 270076<br>15 arteriosclerosis.ti,ab. 12756<br>16 exp rheumatoid arthritis/ 223732<br>17 "rheumatoid arthritis".ti,ab. 171357<br>18 exp diabetes mellitus/1118746<br>19 "diabetes mellitus".ti,ab. 319539<br>20 ("type one diabetes" or "type 1 diabetes").ti,ab. 73651<br>21 ("type two diabetes" or "type 2 diabetes").ti,ab. 229055<br>22 glucose intolerance/ 20380<br>23 "glucose intolerance".ti,ab. 16233<br>24 exp hypertension/ 848245<br>25 hypertension.ti,ab. 651056<br>26 "high blood pressure".ti,ab. 25126<br>27 12 or 13 or 14 or 15 or 16 or 17 or 18 or 19 or 20 or 21 or 22 or 23 or 24 or 25 or 26 2359637<br>28 11 and 27 272<br>29 limit 28 to english language 268
ERIC	ERIC <1965 to June 2022><br><br>1 "health literacy".ti,ab. 522<br>2 exp Knowledge Level/ or exp Health Education/ 34443<br>3 health.ti,ab. 88079<br>4 2 and 311401<br>5 1 or 4 11621<br>6 (online or electronic or digital).ti,ab. 87726<br>7 5 and 6672<br>8 (eHEALS or "ehealth literacy scale").ti,ab. 4<br>9 7 or 8 673<br>10 arteriosclerosis.ti,ab. 7<br>11 arteriolosclerosis.ti,ab. 0

	12 "rheumatoid arthritis".ti,ab. 62<br>13 diabetes/ 1007<br>14 ("Diabetes Mellitus" or "type one diabetes" or "type 1 diabetes" or "type 2 diabetes" or "type two diabetes").ti,ab. 395<br>15 "glucose intolerance".ti,ab. 2<br>16 exp Hypertension/ 395<br>17 (hypertension or "high blood pressure").ti,ab. 461<br>18 10 or 11 or 12 or 13 or 14 or 15 or 16 or 17 1623<br>19 9 and 18 13<br>20 limit 19 to english language 13
CINAHL	(S11 OR S12 OR S13 OR S14 OR S15 OR S16) AND (S10 AND S19) Expanders - Apply equivalent subjects Interface - EBSCOhost Research Databases 37<br>Narrow by Language: - english Search Screen - Advanced Search<br><br>Search modes - Find all my search terms Database - CINAHL<br>S20 (S11 OR S12 OR S13 OR S14 OR S15 OR S16) AND (S10 AND S19)<br>Expanders - Apply equivalent subjects Interface - EBSCOhost Research Databases 38<br>Search modes - Find all my search terms Search Screen - Advanced Search<br>Database - CINAHL<br>S19 S11 OR S12 OR S13 OR S14 OR S15 OR S16 Expanders - Apply equivalent subjects Interface - EBSCOhost Research Databases 405,511<br>Search modes - Find all my search terms Search Screen - Advanced Search<br>Database - CINAHL<br>S18 (((MH "Hypertension+") OR hypertension OR "high blood pressure") AND (S11 OR S12 OR S13 OR S14 OR S15 OR S16)) AND (S10 AND S17) Expanders - Apply equivalent subjects Interface - EBSCOhost Research Databases Display<br>Search modes - Find all my search terms Search Screen - Advanced Search<br>Database - CINAHL<br>S17 ((MH "Hypertension+") OR hypertension OR "high blood pressure") AND (S11 OR S12 OR S13 OR S14 OR S15 OR S16) Expanders - Apply equivalent subjects Interface - EBSCOhost Research Databases Display<br>Search modes - Find all my search terms Search Screen - Advanced Search<br>Database - CINAHL<br>S16 (MH "Hypertension+") OR (hypertension OR "high blood pressure")<br>Expanders - Apply equivalent subjects Interface - EBSCOhost Research Databases Display<br>Search modes - Find all my search terms Search Screen - Advanced Search<br>Database - CINAHL

	S15 (MH "Glucose Intolerance") OR "glucose intolerance" Expanders - Apply equivalent subjects Interface - EBSCOhost Research Databases Display<br>Search modes - Find all my search terms Search Screen - Advanced Search<br>Database - CINAHL<br>S14 (MH "Diabetes Mellitus+") OR ("diabetes mellitus" OR "type 1 diabetes" OR "type one diabetes") OR ("type 2 diabetes" OR "type two diabetes")<br>Expanders - Apply equivalent subjects Interface - EBSCOhost Research Databases Display<br>Search modes - Find all my search terms Search Screen - Advanced Search<br>Database - CINAHL<br>S13 (MH "Arthritis, Rheumatoid+") OR "rheumatoid arthritis" Expanders - Apply equivalent subjects Interface - EBSCOhost Research Databases Display<br>Search modes - Find all my search terms Search Screen - Advanced Search<br>Database - CINAHL<br>S12 "arteriolosclerosis" Expanders - Apply equivalent subjects<br>Interface - EBSCOhost Research Databases Display<br>Search modes - Find all my search terms Search Screen - Advanced Search<br>Database - CINAHL<br>S11 (MH "Arteriosclerosis+") OR Arteriosclerosis Expanders - Apply equivalent subjects Interface - EBSCOhost Research Databases Display<br>Search modes - Find all my search terms Search Screen - Advanced Search<br>Database - CINAHL<br>S10 (MH "Health Literacy") AND (digital OR electronic OR online) OR (eHEALS OR "ehealth literacy scale"
LLISI	S3 S1 AND S2 Expanders - Apply equivalent subjects<br>Search modes - Find all my search terms Interface - EBSCOhost Research Databases<br>Search Screen - Advanced Search<br>Database - Library Literature & Information Science Index (H.W. Wilson) 2<br>S2 arteriosclerosis OR arteriolosclerosis OR "rheumatoid arthritis" OR "Diabetes Mellitus" OR "type 1 diabetes" OR "type one diabetes" OR "type 2 diabetes" OR "type two diabetes" OR hypertension OR "high blood pressure"<br>Expanders - Apply equivalent subjects<br>Search modes - Find all my search terms Interface - EBSCOhost Research Databases<br>Search Screen - Advanced Search<br>Database - Library Literature & Information Science Index (H.W. Wilson) Display

	S1 (DE "Health literacy" OR "health literacy") AND (digital OR electronic OR online) OR (eHEALS OR "ehealth literacy scale") Expanders - Apply equivalent subjects<br>Search modes - Find all my search terms Interface - EBSCOhost Research Databases<br>Search Screen - Advanced Search<br>Database - Library Literature & Information Science Index (H.W. Wilson)
Google Scholar	allintitle: "eHEALS"
